# Supplementary material for: Are carbon emissions trading and green financial instruments synergistic? -Comprehensive quantitative research based on content analysis
Source: PLoS One. 2024 Mar 7;19(3):e0298601. doi: 10.1371/journal.pone.0298601 (PMC10919601; doi:10.1371/journal.pone.0298601)
Supplement: S1 Data — (ZIP) [file pone.0298601.s001.zip › Data/地级市Z_F工作报告.zip!/║╙─╧/┐¬╖Γ╩╨/└┤╫╘╣1⁄2╓┌║┼í╛┬φ┐╦╩2╛▌═°í┐_2019.docx]

各位代表：

现在，我代表市人民政府，向大会报告工作，请予审议，并请各位政协委员和其他列席人士提出意见。

一、2018年工作回顾

过去的一年，面对严峻复杂的经济形势和艰巨繁重的改革发展任务，在省委、省政府和市委的坚强领导下，全市上下高举习近平新时代中国特色社会主义思想伟大旗帜，全面落实党的十九大精神和习近平总书记视察指导河南及开封时的重要讲话精神，坚持稳中求进工作总基调，坚持新发展理念，以高质量发展为根本方向，以供给侧结构性改革为主线，按照 “贯穿一主线一保障，确保两稳定，打好三攻坚，抓实五突破”工作布局，持续保底线、保民生、保运转、优环境、上项目、求发展，较好完成了市十五届人大一次会议确定的目标任务，全市经济社会呈现“稳、进、好”的态势。一是“稳”。全市生产总值突破2000亿元大关，增长7%；一般公共预算收入140.68亿元，增长14.6%；信访总量、事故起数分别下降38.1%、35%；城镇登记失业率控制在2.61%。二是“进”。三次产业结构调整为13.7∶38.9∶47.4，三产比重提高2.5个百分点，服务业拉动经济增长3.5个百分点；新增城镇常住人口7.27万人，城镇化率达到48.9%；土地利用综合改革、国企改革、“放管服”改革、普惠金融改革、人才管理体制改革“五大改革”取得实质性进展。三是“好”。税收突破100亿元大关，占一般公共预算收入的73.1%，高于全省2.5个百分点；单位生产总值能耗下降8.8%；全市居民人均可支配收入增长9.3%，社会大局保持和谐稳定。过去的一年，亮点纷呈。开封海关成功获批，开放平台实现新突破；争取到全国首批黑臭水体治理示范城市、全域普惠金融改革试验区、全省首批促进科技与金融结合试点地区等10多个国家级省级试点；城市“双修”、百城提质、自贸区改革创新、构树扶贫等工作经验在全省全国推广。一年来，我们遇到的困难比估计的大，取得的成绩比预料的好。主要做了以下工作：

（一）抓关键稳增长。注重以稳应变，坚持月度决策机制，从制度设计、政策供给、要素保障等多方面稳住预期、稳定发展。积极扩大有效需求。遵循经济规律，从投资、消费、出口三方发力。实施411个强基础、补短板、惠民生的重大项目，完成投资992.2亿元；提升消费供给质量，全市社会消费品零售总额980.6亿元，增长11%，增速居全省第2位；进出口总值增长53.9%，增幅居全省首位。培育壮大实体经济。落实减税降费政策，为企业降低直接成本18亿元；大力发展非公有制经济，出台三十条具体扶持政策，非公有制经济增加值完成1279亿元，占生产总值比重达63.9%；激发市场活力，全市各类市场主体达到26.7万户，增长17.7%，注册资本额增长23.9%。强化要素保障。搭建政银企对接平台，帮助企业多渠道融资，融资总额突破110亿元；规范土地管理供应机制，全年供应土地1.38万亩，有效保障重点项目用地；持续实施“汴梁英才计划”，招揽各层次专业人才9.8万人。

（二）调结构促转型。深化供给侧结构性改革，把“转型”“升级”“融合”作为主攻方向，推动发展提质增效。加快制造业转型。坚定“四化”目标，推进制造业智能、绿色、技术“三大改造”，总投资650亿元，实施转型发展项目163个，完成“机器换人”项目12个，改造智能化生产线23条，国家绿色示范工厂实现“零”的突破。规模以上工业增加值增长7.7%，奇瑞汽车、开封空分、开封炭素等重点骨干工业企业产值分别增长124%、99.8%、77.1%，带动全市规模以上工业企业利润总额增长28.5%，高于全省12.5个百分点。升级现代服务业。全市服务业增加值完成949.78亿元，增长8.2%，对经济的贡献率达50.6%。央视12次报道开封文化旅游，新增4A级旅游景区2个，全年游客接待量6806.8万人次，旅游综合收入602.2亿元，分别增长16.1%和24.6%。持续“引金入汴”，新引进金融、类金融机构18家，余额存贷比72.8%。以“文化+”战略为引领，汇聚新业态新模式，万达广场、鼓楼里、960非遗文创园（一期）等文商旅综合体建成开放。促进一二三产融合发展。以“五优四化”为重点，深化种养业供给侧结构性改革，新建高标准粮田38.9万亩，粮食总产301.1万吨，粮食安全市长责任制考核全省第3名。加快农业产业化步伐，新增工商注册专业合作社591家，农业产业化龙头企业达到150家，现代农业产业化集群达到19个。

（三）聚合力打攻坚。始终把打好三大攻坚战作为破解瓶颈制约、决胜全面建成小康社会的硬任务，凝聚全市之力，精准推进。防范化解重大风险。坚持底线思维，积极稳妥化解债务存量，严控债务增量。有效防范金融风险，全市银行业金融机构不良贷款率3.18%，低于5%的监管要求。全面防控自然灾害、公共事件、网络信息安全等重点领域风险，有效阻断非洲猪瘟等重大疫情。扎实开展精准脱贫。聚焦“六个精准”，出台脱贫攻坚系列政策措施，累计投入扶贫资金27.94亿元，实施扶贫项目569个，74个贫困村2.79万人标注脱贫，贫困发生率降至1.46%。黄河滩区居民迁建工作取得重大进展。铁腕推动污染防治。深入践行习近平生态文明思想，着力管好大气污染源，严格落实“六控”措施，综合利用约谈、曝光、问责等手段铁腕治污，优良天数全省排名由第三方阵上升至第二方阵，冬季清洁取暖试点工作居国家四部委考核第2名。启动“四水同治”，省控地表水断面水质全部达到省下目标要求，如期完成水源地综合整治任务，集中式饮用水水源地水质达标率保持100%。开展大规模国土绿化工程，完成造林10.3万亩。

（四）勇革新求突破。充分激发改革推动、创新驱动、开放带动、优化环境四个动力，加快新旧动能转换。深化重点领域改革。深入推进国有企业改革，完成市属70户“僵尸企业”处置任务，推行市属国有资本投资运营公司分类管理，建立市场化薪酬和绩效考核制度，被评为国企改革攻坚工作先进省辖市。扎实开展土地利用综合改革，坚持“五统一”，实现土地出让收入150亿元以上。全域推动普惠金融改革，建成普惠金融服务站1898家，到位风险补偿金1亿元，普惠金融贷款余额346亿元。加快“放管服”改革，在全省率先推出“六个一”政务服务规范，行政审批服务事项网上办理实现率、网上可办实现率和一网通办实现率均居全省前列，“互联网+政务服务”工作经验被国务院、省政府专题总结推广。稳步推进人才管理体制改革，在薪酬待遇、子女入学、就医、住房等多方面强化保障，成功引进“双一流”院校硕士研究生134人。市县机构改革进展顺利，生态、财税、教育、卫生、文化、农村、市场监管等领域改革取得新成效。强力推进创新驱动。聚焦“四个一批”，狠抓“四个融合”，中原先进技术研究院、中关村智酷人才与产业创新基地落户开封，战略性新兴产业和高技术产业增加值占规模以上工业增加值比重提升到9.8%，高新技术企业达到62家，创新型龙头企业和“科技小巨人”企业达到22家，国家科技型中小企业突破100家，重点实验室等科技创新平台突破200家。大幅提升开放水平。自贸区开封片区示范引领作用不断增强，电子口岸入网联审优化改革经验向全省复制推广；成功举办中日韩友好城市交流大会、全国中小企业发展大会，新引进省外资金项目246个，实际到位省外资金618.3亿元，益海嘉里、上海均和等一批大项目、好项目落户开封。持续优化营商环境。启动营商环境国际化引领区建设，在全省率先制定营商环境评估指标体系，引入第三方评估机制，定期开展营商环境双月点评。推进“证照分离”“多规合一”以及企业投资项目承诺制，优化各类服务流程297个，压缩各类审批材料984份，外资企业设立审批效率提高1倍多。二号首长

（五）重内涵促统筹。统筹推进乡村振兴战略和城市转型内涵式发展，城乡更趋协调。坚持规划引领。全面启动城市总规修编，高标准完成宋都古城保护与修缮、重点片区城市设计等32个专项规划，编制水生态文明、乡村振兴“1+6”示范带等一系列重点规划。实施乡村振兴战略。研究制定乡村振兴十大行动计划。加强农村人居环境整治，推广农村垃圾清运市场化运营，修建农村公厕、户厕1.8万座，建成“四好农村路”179公里，超额完成农村危房改造任务，创建美丽宜居乡镇15个、美丽宜居村庄50个，西姜寨乡获得“河南十大最美乡村”称号。推进百城建设提质。实施重大基础设施项目336个，累计完成投资156.5亿元。机西高速建成通车，与连霍高速、郑民高速、大广高速、日南高速形成绕城闭环；开港大道、魏都路东延等11条道路竣工通车；新增停车场25个、停车泊位4967个；入选河南省“公交优先”示范市创建城市。实施城市“双修”，完成大梁路、曹门大街等街景整治，完成环城墙公园七期、复兴坊历史风貌区修缮整治；全省百城建设提质工程暨文明城市创建工作推进会在我市成功召开，创建全国文明城市、国家生态园林示范城市等“五城联创”工作取得重大进展。

（六）惠民生增福祉。着力破解民生“八需八难”，累计投入民生资金263.67亿元，占财政支出的71.5%，省、市民生实事全部落地见效。稳定扩大就业。建成国家级高技能人才培训基地，完成各类就业培训14.8万人次，城镇新增就业7.18万人，农村转移就业3.23万人，返乡下乡创业带动就业30.4万人。发展社会事业。加强社会保障，全民参保登记入库537万人。推出教育“十项大礼包”，公开招聘教师2338名，新建、改扩建幼儿园16所、中小学15所，新增学位2.2万个；圆满完成“全面改薄”任务；支持河南大学“双一流”建设取得实质性进展。持续落实分级诊疗，医疗服务价格动态调整机制逐步完善，全市人均基本公共卫生服务经费由50元提高至55元。建成开封康复医院。新建全民健身工程134处，城市社区健身设施覆盖率达到100%。“六馆一城”建成并免费对外开放；村级综合性文化服务中心建成率达96.1%，居全省前列。改造提升72所敬老院，新建养老机构6家，新增养老床位1000余张。推动社会治理创新。加快基层社会治理体系“一中心四平台”建设，社区治理和服务能力建设经验在全国推广。加强安全生产等重点行业领域专项治理，法治信访“一中心一机制”全面推开，“宋都调解”纠纷化解方式得到中央政法委充分肯定，小餐饮食品安全监管治理做法被人民日报刊发。“扫黑除恶”战果丰硕，群众满意度全省排名第一，全市人民获得感、幸福感、安全感持续增强。

不断加强国防教育和国防后备力量及人民防空建设，支持军队改革发展，推进军民融合深度发展，驻汴解放军、武警部队、预备役人员、民兵为地方发展做出新贡献，双拥工作持续深化。民族宗教工作创新推进。妇女儿童、残疾人、红十字、慈善等事业健康发展。统计、侨务、港澳、对台、史志、档案、文物、气象、地震、社科研究、援疆等工作取得新成绩。

一年来，我们自觉接受人大法律监督、政协民主监督和社会舆论监督，认真办理人大代表议案、建议和政协提案，积极支持民主党派、工商联和无党派人士参政议政。加强法治政府建设，制定2部政府规章，提请市人大常委会制定1部地方性法规，积极推进依法行政；强化审计监督，驰而不息纠正“四风”，政府自身建设和治理能力不断提升。

各位代表！过去的一年，看似波澜不惊，实则挑战严峻。在工作中，我们更加深切地体会到：要战胜困难、取得胜利，必须树牢“四个意识”，坚定“四个自信”，坚决做到“两个维护”；必须树牢居安思危、防患未然的底线思维和忧患意识，为高质量发展筑牢基础；必须树牢追求卓越、勇创一流的竞争意识和出彩意识，确保各项工作走在前列。实践证明，无论形势多么复杂、挑战多么严峻、任务多么艰巨，只要有习近平同志为核心的党中央掌舵领航，以逢山开路、遇水架桥的魄力和胆识，凝聚起550万开封人民的磅礴力量，就一定能推动开封各项事业向更高质量发展，就一定能取得让人民满意、各界认同的发展成就。

过去一年的成绩来之不易，离不开省委、省政府和市委的坚强领导；离不开全市人民以习近平新时代中国特色社会主义思想为指引，拼搏进取、务实重干；离不开历届领导班子打下的坚实基础；离不开市人大、市政协有效监督和大力支持；离不开驻汴单位的共同参与。在此，我代表市人民政府，向工作在全市各条战线上的广大工人、农民、知识分子、干部，向驻汴人民解放军指战员、武警官兵、公安民警，向各民主党派、工商联、无党派及社会各界人士致以崇高的敬意！向所有关心、支持开封发展的同志们、朋友们表示衷心的感谢！

看到成绩的同时，我们也清醒认识到，我市经济社会发展中还面临不少困难和问题：实体经济依然困难，中小微企业“融资难、融资贵”问题较为突出；新旧动能转换不快，创新型龙头企业还不多，新业态、新模式较少，产业结构不优的状况尚未根本改变；资金、土地、人才等制约瓶颈依然存在，全市固定资产投资、民间投资增速放缓；民生还有不少短板，生态环境仍需进一步改善，就业、就学、就医等公共服务还不能满足群众高质量需求；一些领域风险不容忽视，非法集资、问题楼盘等不稳定因素尚未完全化解。同时，一些干部还存在观念落后、作风不硬、标准不高、能力不足等问题，一些领域不正之风和腐败问题时有发生。对这些问题，我们一定高度重视，认真加以解决，努力把各项工作做得更好更扎实。

二、2019年工作安排

2019年是中华人民共和国成立70周年，是习近平总书记视察指导河南及开封5周年，也是决胜全面建成小康社会的关键之年，做好今年的工作意义重大。

今年政府工作要认真落实市委十一届八次全会要求：以习近平新时代中国特色社会主义思想为指导，全面贯彻党的十九大和十九届二中、三中全会精神，持续落实习近平总书记视察指导河南及开封时的重要讲话精神，按照中央经济工作会议和省委十届六次、八次全会部署，坚持稳中求进工作总基调，坚持新发展理念，坚持以人民为中心的发展思想，坚持以供给侧结构性改革为主线，贯彻巩固、增强、提升、畅通的方针，统筹推进稳增长、促改革、调结构、惠民生、防风险、保稳定，坚持“1234”总体工作思路，坚持“贯穿一主线一保障，确保两稳定，打好三攻坚，抓实五突破”工作布局，保持经济运行在合理区间，进一步稳就业、稳金融、稳外贸、稳外资、稳投资、稳预期，提振市场信心，增强人民群众获得感、幸福感、安全感，保持经济持续健康发展和社会大局稳定，为全面建成小康社会收官打下决定性基础，在以党的建设高质量推动经济发展高质量、新时代开封更加出彩进程中迈出坚实步伐，以优异成绩庆祝中华人民共和国成立70周年。

今年经济社会发展的主要预期目标：继续保持“三个同步”“三个高于”，全市生产总值增长7.2%—7.7%，规模以上工业增加值增长7.5%左右，全员劳动生产率增长7%，一般公共预算收入增长10%左右，税收占一般公共预算收入比重达到72%以上，固定资产投资增长8.5%左右，社会消费品零售总额增长11%左右，居民消费价格指数涨幅控制在3%左右。更加突出优化经济结构。服务业增加值占生产总值的比重达到47.5%，质量效益明显提升。更加突出创新发展。研究与试验发展经费投入强度达到1.5%，战略性新兴产业增加值、高技术产业增加值占规模以上工业增加值比重分别达到6.5%、4.2%，技术合同成交额增长20%，高新技术企业达到75家，每万人发明专利拥有量达到1.8件。更加突出协调发展。常住人口城镇化率提高1.7个百分点，人均预期寿命持续提高，九年义务教育巩固率达到96%，医疗卫生、文化、体育等社会事业全面进步。更加突出绿色发展。单位生产总值能耗下降3%以上，全市PM2.5平均浓度、PM10平均浓度、空气优良天数比例、主要污染物排放量等环保指标完成省下达目标。更加突出开放发展。进出口总额增长10%，实际利用外资增长3%，实际利用省外资金增长5%，营商环境进一步优化。更加突出共享发展。居民人均可支配收入增长8%左右，44个贫困村退出贫困序列，3.35万农村贫困人口实现脱贫，城镇新增就业6万人，城镇调查失业率、城镇登记失业率分别控制在5.5%以内、4%以内。确定以上总体要求和预期目标，更加凸显新发展理念，有利于摆脱惯性思维、路径依赖，把工作重点放在推进高质量发展上来。

工作中，要着力把握“五大原则”：一是坚定走准发展方向。必须坚持习近平新时代中国特色社会主义经济思想，走准走稳高质量发展路子，才能方向不偏、行稳致远。二是牢牢把握城市定位。市委明确“贯穿一主线一保障，确保两稳定，打好三攻坚，抓实五突破”工作布局，提出“两城两都一支点”发展定位，找准了路径方向，必须牢牢把握、贯彻执行。三是紧紧抓住工作重点。必须认真领会党中央国务院和省委省政府会议精神，始终坚持以供给侧结构性改革为主线，全面贯彻落实“八字方针”，深刻把握投资、消费和出口关键支撑，发挥改革开放创新驱动作用，推动重点领域、重大事项取得新突破。四是坚决防范重大风险。必须发扬斗争精神，提高斗争本领，重点关注企业经营风险、问题楼盘风险、非法集资风险、互联网金融风险、安全生产风险、信访稳定风险、社会治安风险等，打好化险为夷、转危为机主动战。五是切实强化民生保障。必须坚持“尽力而为、量力而行，完善制度、守住底线”原则，以破解民生“八需八难”为总抓手，以完成省、市民生实事为“硬任务”，紧盯群众最急最需最忧最盼，不断加大民生投入，让老百姓感受到发展的温度、幸福的质感。

同时，要抢抓“五大机遇”：一是抓住宏观经济政策机遇。宏观政策将强化“逆周期调节”，只要充分利用政策红利，聚焦关键领域扩大有效投资、充分释放消费潜力、积极培育外向型经济，高质量发展的支撑就会更加坚实。二是抓住高质量发展的机遇。省委、省政府已研究出台一系列促进经济高质量发展的政策措施，对于我市加快转变发展方式、优化经济结构、转换增长动能更具有推动作用。只要坚定发展信心，贯彻新发展理念，充分发挥优势，就一定能在高质量发展上大有作为。三是抓住重大战略区位优势机遇。“三区一群”等重大国家战略机遇叠加聚集开封，只要以更加积极的态度抢抓这些重大机遇，超前谋划、积极对接、主动融入，就一定能实现跨越发展。四是抓住深化制度改革机遇。改革开放仍需跋山涉水。只要勇于改革开放，坚定不移聚焦营商环境国际化引领区建设，持续深化“放管服”、土地利用、国资国企、普惠金融、人才管理体制等重点领域改革，就一定能全面建设宜居宜业宜游环境，将开封打造成为最具吸引力的投资热土。五是抓住自身发展带来机遇。近年来，开封发展步伐越来越快，基础更加坚实，潜力优势正在聚集释放，尤其是四大班子精诚团结，县区、部门干劲越来越大，政通人和、干事创业的氛围已经形成。只要我们乘势而上，不松劲、不懈怠，就一定能够创造开封更加出彩的明天。

今年重点抓好八个方面的工作：

（一）持续打好三大攻坚，牢牢守住高质量发展底线。坚持把“三大攻坚战”作为首要政治任务、必须牢牢守住的重大底线，坚决打好打赢。

防范化解风险。按照“八个一批”方式，防范化解政府债务风险，依法规范政府举债行为，有效控制债务率。防范金融风险，严厉打击非法集资等违法违规金融活动，严防金融机构出现操作风险、信用风险和流动性风险。防范社会风险，压实安全生产责任，确保实现“一杜绝两下降”目标。抓好重点群体信访维稳，推进法治信访“一中心一机制”建设，开展积案化解攻坚，妥善解决拖欠工程款、农民工工资等群众切身利益问题；加强网络综合治理，推进依法治网。完善自然灾害应急处置机制，有效防控非洲猪瘟等疫情。持续深化扫黑除恶专项斗争，严厉打击黄赌毒、盗抢骗、网络传销等各类犯罪，维护社会治安大局稳定。

推进精准脱贫。严格贯彻“六个精准”“五个一批”要求，加大深度贫困村、特殊贫困群体脱贫攻坚力度，重点解决好实现“两不愁三保障”面临的突出问题。继续打好“四大硬仗”、开展“五大行动”、实施“四大工程”，持续推进“六业扶贫”，深化行业扶贫，确保所有贫困村出列、3.35万农村贫困人口稳定脱贫。着力破解贫困人口返贫、贫困村集体经济发展等问题，切实提高脱贫攻坚质量。全面完成祥符区、兰考县5812户20737名黄河滩区群众搬迁和旧村复垦工作，真正让老百姓搬得出、稳得住、能发展、可致富。

抓好污染防治。深入实施经济结构提质、生态功能提升、国土绿化提速、环境治理提效“四大行动”。打好蓝天保卫战，调整优化能源、产业、交通、用地四大结构，持续强化扬尘污染管控，严控秸秆垃圾焚烧，推进机动车和低空面源污染防治，开展产业集聚区和工业园区企业绿化、净化、美化专项整治。打好碧水保卫战，加强饮用水水源地保护，全面落实河长制湖长制，开工建设北区污水处理厂、运粮河污水处理厂和20个黑臭水体治理项目，完成东区、西区污水处理厂扩容。打好净土保卫战，严格执行《土壤污染防治法》，加强建设用地风险管控，做好重金属污染防控、重点行业企业用地调查、固体废物和危险废物规范化管理工作，开工建设3个静脉产业园。

（二）积极扩大有效需求，保持经济发展稳中有进。千方百计瞄准“有效”，精准发力，扩大投资，推进内需外需稳定增长。

扩大有效投资。开展“项目推进效率年”活动，聚焦产业转型、文化旅游、民生服务、生态环保、基础设施、现代农业等6大领域，重点实施“三个100”工程，确保签约落地、新开工建设、竣工建成重大项目各100个以上。落实好重大项目推进“六项机制”和首席服务官制度，“一企一策”破解难题，实现项目建设接续推进。拓宽融资渠道，规范运用引导基金、PPP、BOO等多种模式，积极争取国家、省项目资金支持。放宽市场准入，激发民间投资活力，确保民间投资增速高于固定资产投资增速。

释放消费潜力。以更大气魄建设国际文化旅游名城，打造中原现代服务业之都。落实个人所得税专项附加扣除政策，重点激活文化旅游、房地产、汽车家居、教育体育、健康养老等消费热点，优化消费产品供给。深入开展旅游环境综合整治，完善旅游设施，提升全域服务品质。升级改造一批开放式街区和城市综合体，新建一批高星级酒店、特色精品酒店，择优培育评定一批金宿级、银宿级旅游民宿。持续办好“一带一路”城市旅游联盟年会及菊花文化节、清明文化节等重大节会，策划包装郑开国际马拉松赛、足球联赛等赛事活动，办好开封市第十届运动会。严厉打击侵害消费者权益行为，改善消费环境，让消费者“能消费”“愿消费”“敢消费”，力争全市三产比重提升2个百分点。

加强对外贸易。加快推进综合保税区申建工作，统筹规划建设文化艺术品保税仓、医疗器械保税仓，建成上海均和集团国际贸易结算中心，新建均和全球供应链总部基地及跨境贸易综合体。主动融入“一带一路”，规划建立港区联动空运货物开封服务中心、开封跨境电子商务发展中心，支持外贸企业建立“海外仓”。加强与曼彻斯特市等国际城市交流合作，组织优秀企业、优质产品“走出去”，提高经济外向度。

（三）推进产业转型升级，促进实体经济做强做优。突出发展先进制造业，实施智能制造引领、重点技术攻坚、传统产业提质、新兴产业培育“四大行动”，推动先进制造业与现代服务业、现代农业、科技创新融合发展。

加快制造业转型升级。强化政策引导，持续推动16个重点产业转型发展，深入实施传统制造业“三大改造”，大力开展“质量标杆进企业”活动，弘扬劳模精神和工匠精神，支持企业增品种、提品质、创品牌。支持8家企业创建绿色示范工厂；谋划实施一批技术改造项目，完成技改投资150亿元以上；加快工业智能化改造，建设智能化示范生产线30条，力争省级智能工厂达到4个、智能车间达到8个。建立支持工业经济“四个50”机制，紧盯50家龙头企业、50家“双高”企业、50家困难企业和50家动态入库企业，搞好服务、破解难题、促进发展。加快晋开、东大等工业企业“退城入园”，促进产业集聚区提质增效。

提升重点产业链水平。聚焦“8+1”产业链，健全“六个一”培育机制，着力补链、延链、强链，打造主导产业优势明显、大中小企业协同配套的优势产业集群。提升汽车及零部件产业链，加快推进奇瑞汽车30万台整车扩建项目，开展配套企业招商，着力引进新能源汽车、汽车电气等配套项目。提升装备制造产业链，引导中联重科、茂盛机械等制造业企业研发新产品、开拓新市场，推动开封空分、东京空分等空分企业链式集群发展。提升精细化工产业链，做大做强杞县聚甲醛等项目，支持精细化工产业集聚区争创省级高新区，引导化工产业向研发领域延伸、向精细化方向发展，创建绿色园区。提升医药和医疗器械产业链，支持生物制药、医疗器械、医疗服务等行业发展。提升纺织服装产业链，引导企业加大服装设计、品牌策划环节投入，向高附加值方向转型。提升家居产业链，加快恒大家居联盟产业园、碧桂园现代筑美家居产业园等项目建设，推动家居产业链向智能家居、整体家装方向延伸。提升新材料产业链，加快开封炭素二期工程建设，启动炭素新材料产业园规划建设，建成超高功率石墨电极生产线和研发中心；支持铝型材和铝精深加工产业发展，大力招引铝箔、交通用铝、航空用铝等新材料研发制造企业。提升农副产品加工产业链，推动益海嘉里、河南省大蒜现货交易中心等项目建设，新建一批畜禽、花卉、果蔬、木材等特色高效农产品生产加工基地，打造全链条、全循环、高质量的农副产品加工产业化集群。提升文化旅游产业链，充分发挥文化旅游资源优势，积极创建国家全域旅游示范城市，促进旅游业与传媒、养生、会展等行业深度融合，推动“文化+”向“高端要素+文化”拓展，推进清明上河园三期、朱仙镇二期、小宋城二期、960·乐在宋潮等文旅项目建设，深度挖掘开封文化IP资源，打造一批独具开封特色的文化品牌，让开封文化走出去、有活力。

积极培育新兴产业。按照河南省培育发展“10+8”新兴产业集群要求，充分发挥自贸区、经济技术开发区、电子商务示范基地等国家级园区优势，大力发展新经济、新业态、新模式。着力发展数字经济，编制完成《开封市数字经济发展规划》，推进易华录中原数据湖等智慧城市项目建设，推动大数据与实体经济深度融合，着力壮大智慧产业、数字经济体量。做强电子商务和物流产业，探索“电商产业园+物流园”融合发展新模式，建设县乡村三级电商物流网络体系。积极培育绿色经济、总部经济、足球产业、金融服务业等新业态，逐步形成拉动经济增长的新动能。

加快一二三产融合。加快转变农业发展方式，构建现代农业产业体系，形成“一产接二连三”的互动型、融合型集群发展模式。认真贯彻落实粮食安全生产责任制，新建高标准粮田30万亩，持续巩固粮食生产能力。大力支持农业龙头企业发展，建设一批与龙头企业有效对接的生产基地，实现农业生产经营专业化、标准化、规模化。推进“五优四化”，发展高效种养业和绿色食品业，叫响杞县大蒜、开封西瓜、兰考蜜瓜等农产品品牌，支持更多农产品申报国家地理标志。扶持奶牛肉牛肉羊产业发展，创建国家级和省级稻渔综合种养示范区、水产健康养殖示范场。推动农业与文化旅游、教育体育、健康养老等产业互通互融，打造一批特色鲜明的田园综合体。

（四）深化改革开放创新，充分释放发展动力活力。深刻把握高质量发展的关键，充分激发要素资源潜力，持续为经济社会发展注入新动能。

深化重点领域改革。推进机构改革，3月底前基本完成市县区机构改革。深化土地利用综合改革，坚持“五统一”，科学制定土地供应计划，保障安置房建设用地，完成补充耕地1.5万亩。深化国企改革，稳妥解决市属70户“僵尸企业”处置后续问题，积极帮扶下岗困难职工；加快实现从管资产向管资本转变，做好企业清产核资，完善现代企业制度，全力支持国有企业混合所有制改革和上市发展。全面推进普惠金融改革试验区建设，加快建设“一平台四体系”，深化“普惠通”APP与金融机构合作，为中小微企业、工商个体、创客群体、贫困户、普通市民等提供更加便捷、多元化的金融服务。同时，抓好财税、教育、科技、文化、社保等改革。

大力开展招商引资。开展“精准招商服务年”活动，按照“门槛要高、条件要优”原则，围绕“8+1”产业链，招引一批国内外500强、上市公司等龙头企业；围绕培育新业态，招引企业总部、专业咨询、会展经济、赛事策划等新兴产业；围绕外向型经济，招引国际贸易、国际物流、国际金融等外向型企业。创新招商方式，实施专业招商、定点招商、商会招商、以商招商、小分队招商等；注重节会招商，筹备好清明文化节经贸活动、菊花文化节系列招商活动，组织参加第十一届中国中部投资贸易博览会、第十三届投洽会等系列经贸活动，力争引进5亿元以上项目55个。落实好招商引资“六项机制”和“五个一”跟踪服务机制，切实提高项目履约率和资金到位率，严格兑现政府承诺。

推进创新驱动发展。主动融入郑洛新国家自主创新示范区，加快郑开双创走廊建设，支持开封市大学科技园创建省级大学科技园，全力打造全省“双创”示范基地。提高财政投入效率，引导企业增加研发投入，鼓励企业与高校、国家级科研机构共建研发平台，支持奇瑞汽车研究中心、环保铝型材加工研究院、开炭新材料设计研究院等研发机构建设，让更多科研成果在开封开花结果。加快促进科技与金融结合试点地区建设，积极探索科技金融开封模式。新增省市级工程技术研究中心20家、市级重点实验室5家以上、创新创业孵化平台5家以上。力争培育创新型龙头企业达到10家、“科技小巨人”企业达到20家、国家科技型中小企业入库100家以上。

（五）加快城乡融合发展，形成协调发展新格局。坚持把城乡作为一个整体，优化国土空间布局，统筹推进新型城镇化和乡村振兴战略，实现区域协调发展新突破。

深入实施乡村振兴战略。围绕农业强、农村美、农民富，加快推进产业、人才、文化、生态、组织“五个振兴”。科学编制全市及各县区乡村振兴五年规划，高标准推进10个省级乡村振兴示范乡镇和31个市级乡村振兴示范村建设，完成乡村振兴“1+6”示范带阶段性任务。持续深化农村改革，推动乡村土地全域综合整治试点工作，盘活农村土地资源，吸引更多优质资本下乡，壮大农村集体经济，增加农民收入。持续实施农村饮水安全巩固提升工程。开展农村人居环境“千村示范、万村整治”工程，确定100个示范村、1000个整治村，突出垃圾治理、污水处理、厕所革命，补齐农村基础设施短板。深入推进移风易俗，培育优良家风、文明乡风和新乡贤文化，实现农村人居环境与文明素质双提升。

全域推进百城提质工程。坚持城市规划、建设、管理、经营“四个高质量”，做好以水润城、以绿荫城、以文化城、以业兴城“四篇文章”，促进城市由外延扩张转向内涵提质。把规划作为城市建设的“第一粒扣子”，汲取先进城市规划理念，高标准做好新一轮城市总体规划、土地利用总体规划修编工作，完成城市“双修”、生态水系、综合交通、乡村振兴等专项规划编制和重点片区城市设计。实施棚户区改造三年行动计划，加快推进“1+7+N”棚户区改造项目，实现4个棚户区改造遗留项目征收清零；高标准做好5000亩安置房建设用地规划选址和建设工作。着力提升城市综合承载能力，完成老旧小区整治改造任务30个以上。完成拆除违章建筑三年行动计划目标。以交通治“堵”、环境治“污”、市容治“脏”、服务治“差”为重点，强化城市精细化管理，扎实开展全国文明城市、国家生态园林示范城市等“五城联创”。加强现代公共文化服务体系建设，完善“六馆一城”，推动县级图书馆、文化馆、博物馆全部达到部颁三级标准，村级综合性文化服务中心实现全覆盖，大力倡导全民阅读，提升城市品质。

加强古城保护和利用。深入推进“五个全域”，继续实施城市“双修”，加快城墙贯通工程、故宫文物数字化馆开封分馆等项目建设。加快开封古城保护立法，争取设立宋都古城保护修缮专项基金。实施历史文物保护性修缮，强化物质文化遗产保护和非物质文化遗产传承创新，做好古州桥汴河遗址考古发掘工作，保护好文化遗存、民俗民风，争创国家文物保护利用示范区，延续古城历史文脉。

推进郑汴港深度融合。积极融入郑州大都市区建设，持续推进郑汴一体化“533”工程，高标准编制完成郑开双创走廊、沿黄生态带、大运河文化带等专项规划。支持城乡一体化示范区实施“大提升、大招商、大建设”行动，做好概念规划及重点区域城市设计，开工建设西湖二期工程。全面推进自贸区开封片区“一心四谷两港”建设，推动中央商务中心成规模、健康乐谷出形象，大力发展文化创意、金融服务、现代物流等新兴服务业，做实中关村智酷人才与产业创新基地、绿地中部创客天地等创新载体，力争新打造税收超亿元楼宇1个、超千万楼宇6个，新入驻企业1500家以上。推进国家产城融合示范区土地流转、收储以及生态廊道建设工作，发展临港经济。强化区域协调发展，持续提升各区综合实力，统筹推进东部新城、北部片区、南部片区基础设施和产业项目布局。

（六）推进实施重大工程，强化基础支撑能力。持续以“八网”建设为重点，久久为功，善作善成，提升城市服务功能。完善路网。畅通“五环九横十二纵”城市路网，畅通各县、祥符区与市区通道。加快郑开城际铁路延长线、郑民高速杏花营收费站、连霍高速开柳路龙亭站出入口改扩建，开尉路北延及开尉路慢行系统等工程建设。完善水网。加快《开封市水生态文明建设暨四水同治专项规划》编制工作，做好南水北调入汴、涡河治理、王庵引黄调蓄工程前期工作；完成“一渠六河”、涧水河综合治理工程，全面启动水系一期工程；实施惠济河流域生态治理、马家河综合治理、运粮湖引黄调蓄工程等十大重点水利工程，加快赵口灌区二期、大运河疏浚等工程建设，打造亲水宜人水生态格局。完善绿网。积极创建国家森林城市，实施国土绿化十大工程，启动市区防护林建设，完成造林15.6万亩。对夷山大街等8条道路和环城墙、水系进行高标准绿化改造提升，完成环市区连霍、大广、郑民、机西4条高速和开港大道两侧绿化工作，全市建成区绿地率达到36%。广泛开展绿色单位、绿色村镇等评选活动，努力实现三季有花、四季常青。完善热网。持续推进北方地区冬季清洁取暖试点城市项目建设，提高热源供给，新敷设供热主管线30公里，完成30座换热站升级改造，实现新增入网面积500万平方米，城区集中供热普及率达到90%。完善气网。新敷设中压管网70公里，新增用气5万户，确保城区燃气气化率达到97%以上，县城燃气气化率达到75%以上。持续增强气源保障，新建150立方液化天然气储罐2个。完善电网。建设35千伏及以上电网工程21项，新增变电容量1023兆伏安，线路长度352.84公里。完善云网。推动智慧开封建设，进一步整合政务云、旅游云、交通云等数据资源，实现大数据资源共建共享。继续实施“宽带开封”战略，确保城市家庭宽带接入能力达到100兆比特每秒以上，积极争取5G网络试点。完善管网。完善地下综合管廊规划，新开工建设地下管廊3公里以上，推进海绵城市建设，提升防汛排涝能力。

（七）深入优化营商环境，持续增强发展软实力。强化营商环境国际化引领区建设，持续巩固“极简”审批、“极速”效率、“极优”服务、“极严”约束成效，全面激发活力。

优化政务环境。持续深化“放管服”改革，巩固扩大“一网通办”前提下“最多跑一次”改革成果，加快“马上办、网上办、就近办、一次办”智能化改革，按照“三级十同”标准，不断完善并联审批模式，实现审批时间压减一半以上。完善优化营商环境评估指标体系、第三方评估和双月点评制度。严格落实国家减税降费政策和我市促进非公有制经济高质量发展“三十条”政策措施，对小规模纳税人“六税两费”减征50%；健全市县两级领导干部联系企业家制度，深化“万名干部进万企”活动，构建亲清新型政商关系。

优化人才环境。坚持引进外部人才和培育本地人才并重，落实“汴梁英才计划”。鼓励大众创业万众创新，用好用活双创平台，让人才有用武之地；推动政府公务人员与金融机构、国有企业等单位人员双向挂职锻炼，畅通人才成长通道；实施“千名精英学子留汴工作计划”，出台人才安居乐业实施办法，切实解决住房、子女教育等实际问题，让人才留得住、能发展。全力支持河南大学“双一流”建设，办好开封大学和开封文化艺术职业学院，加快职教园区发展，为高质量发展提供更加充裕的人才支撑。

优化金融环境。强化金融政策统筹，加快整合信用资源，逐步构建全社会信用体系。实施“引金入汴”工程，推动中原国际绿色（产品）交易中心、嘉兴南湖金融服务有限公司入驻开封，吸引更多金融、类金融机构在开封设立分支；加强政银企定期会商，为在汴企业提供股权融资、债券、基金、融资租赁、资产证券化等多元化融资渠道，发展绿色金融，解决企业融资难题。支持清明上河园主板上市，力争在资本市场直接融资取得新突破。

（八）聚焦八需破解八难，切实增进民生福祉。持续实施省、市民生实事，制定2019年破解民生“八需八难”百项任务清单，精心做好民生工作。聚焦高质量就业需求，开展就业创业“四大工程”，支持返乡下乡创业，确保新增城镇就业6万人，登记失业率控制在4%以内。聚焦便捷出行和停车需求，加强公共交通、停车场、慢行交通、旅游交通等建设，加快断头路打通和交通路口渠化改造；新建智慧城市二期1000路视频探头，推广智慧停车系统，新建公共停车泊位4000个；优化调整公交线路20条，新增公交专用道20公里，建成芦花岗公交场站；落实“百县通村入组工程”和“万村通客车提质工程”，新改建农村公路120公里以上，具备条件的行政村通客车率达到100%。聚焦就近就便入学需求，坚持教育优先发展战略，提高教育教学质量，促进基础教育均衡发展；实施优质教育资源倍增工程三年行动计划，全面落实教育“十项大礼包”，推进优质教育资源合理配置；探索启动中小学课后延时服务机制；新建、改扩建中小学（含农村寄宿制学校）49所、幼儿园20所，普惠性幼儿园覆盖率达到80%；提高教师工资待遇，改善工作生活条件，让教师有礼遇、受尊重。聚焦高水平就医和养老需求，深入推进综合医改，强化“四医”联动，实施“健康素养促进行动”，做好传染病、慢性病防控；完善医疗服务体系，建成2个区域医疗中心，加快市中心医院新院区等十大医疗卫生项目建设，开工建设国家中医药传承创新工程，持续加强基层“中医馆”建设，畅通市县乡三级急救绿色通道；全面建立门诊统筹制度，提高基本医保、大病保险、困难群众大病补充医保保障水平；继续提高城乡居民基础养老金最低标准，继续调增企业和机关事业单位退休人员基本养老金；加快城市社区养老服务设施建设，推动医养结合，满足多层次养老需求。聚焦便捷办事需求，高质量推进“一村（格）一警”，高标准建成“一中心四平台”，推动“互联网+政务服务”向基层延伸，实现全市乡镇（街道）便民服务规范化。聚焦“舌尖上的安全”需求，全面落实“四个最严”“四有两责”要求，持续深化“食药环”专项整治，加强食用农产品交易市场建设和管理，切实强化餐饮、疫苗等食品药品安全监管；建设餐厨废弃物资源化利用、无害化处理中心，完成改造中小学及托幼机构食堂、中型以上社会餐饮场所“互联网+明厨亮灶”1600家。聚焦群众居住需求，加快建立多主体供给、多渠道保障、租购并举的住房制度，争创全国住房租赁市场发展试点城市，基本建成公租房799套，竣工791套；全面完成“四类重点对象”农村危房改造任务；坚持“房住不炒”定位，因城施策、分类指导，推动房地产市场平稳健康发展。聚焦清洁宜居环境需求，完善提升城市道路、照明、排水、污水处理等市政设施，城市主次干道机械化清扫率保持100%。推进垃圾分类处理，推广市场化保洁模式，实现农村生活垃圾收运处置体系全覆盖，加强农村生活污水治理，完成农村户厕改造31.2万户。

加强国防教育、国防动员和国防后备力量建设，支持国防和军队改革，支持驻汴解放军、武警部队、预备役部队建设，深入开展双拥共建，加强退役军人服务保障体系建设，巩固军政军民团结。全面贯彻党的民族政策和宗教工作基本方针，促进民族团结、宗教和睦。支持工会、共青团、妇联等人民团体更好发挥桥梁纽带作用，重视各民主党派、工商联、无党派人士履行职能、发挥作用。做好外事、侨务、港澳、对台工作。加强统计、审计、史志、档案、气象、地震、邮政、社科研究、援疆等工作。

三、加强政府自身建设

新时代、新形势对政府工作赋予新的使命、提出新的要求。各级政府将进一步树牢学的导向、干的导向、严的导向，努力建设人民满意的政府，不辱时代使命、不负人民重托。

（一）以实际行动维护以习近平同志为核心的党中央定于一尊、一锤定音的权威。坚持把党的政治建设摆在首位，牢固树立“四个意识”，坚定“四个自信”，坚决维护习近平总书记党中央的核心、全党的核心地位，坚决维护党中央权威和集中统一领导，真正把“两个维护”内化于心、外化于行，听从党中央号令，紧跟党中央步伐，确保政令畅通、令行禁止，确保习近平总书记重要指示批示和党中央决策部署贯彻落实不走样。加强机关政治建设，深入领会“一个带头、三个表率”要求，弘扬忠诚老实、公道正派、实事求是等价值观，建设一支让党组织放心、让人民群众满意的公务员队伍。

（二）全面建设依宪施政、依法行政的法治政府。弘扬法治精神，深入推进全民普法，让遵纪守法者扬眉吐气，让违法失德者寸步难行。坚持依法决策，认真落实重大决策法定程序，制定《城乡规划管理办法》，提请市人大常委会制定《中小学校幼儿园规划建设条例》《扬尘污染防治条例》，推动市直管公房管理、既有多层住宅增设电梯、餐厨废弃物管理、供水管理、水上交通安全管理、消防管理等立法工作。推进政务公开，开门接受人大法律监督、工作监督，自觉接受政协民主监督，主动接受监察机关监督及社会舆论监督，认真办理人大代表议案建议，及时回应社会关切。加强诚信政府建设，新官要理旧账，做到言必信、行必果。

（三）提高能力素质、锤炼过硬本领。开展“学习的革命”，鼓励到先进地区学习经验，更新观念、开阔视野，以更大力度冲破思想禁锢、摆脱路径依赖，加快形成与岗位相匹配的政治素质、理论功底、领导能力。牢固树立策划规划、资本运作、经营城市等理念，以思维方式的转变推动领导方式、工作方式和发展方式转变。牢固树立精品意识，提高标准、超越自我、追求卓越，以工作高质量推动发展高质量。提高专业能力，干一行学一行，钻一行精一行，注重在推进重点工作、解决重大矛盾、完成重要任务中积累经验、增长才干，成为驾驭复杂形势、解决实际问题的行家里手。

（四）主动扛起接续奋斗、闯关夺隘的责任担当。以对发展负责、对历史负责、对人民负责的态度，始终保持锐意进取、永不懈怠的精神状态，敢于承担风险、迎难而上，勇于挑最重的担子、啃最硬的骨头。大力弘扬焦裕禄同志“三股劲”，推动干部沉下去发现问题、静下心研究政策、出真招破解难题。进一步用好干部激励和容错纠错等制度，坚持严管与厚爱结合，理解基层工作的难处，关心基层干部生活，旗帜鲜明为敢担当者担当、为敢负责者负责，让干事的干部有盼头，让成事的干部有奔头。

（五）时刻筑牢清正廉洁、拒腐防变的思想防线。牢固树立以人民为中心的发展思想，既“身入”基层，又“心到”群众，走好新形势下的群众路线。认真履行全面从严治党主体责任和“一岗双责”，大力开展反腐倡廉，严格执行“八项规定”及实施细则，驰而不息纠正“四风”。健全行政权力监督制约机制，全面加强行政监督、执法监督、纪律监督和审计、财政监督，习惯在巡视、巡察、监督和考核下开展工作。严格执行招投标制度和政府采购制度，用好公共资源交易平台，加强“三公”经费管理，一般性支出压缩5%以上。

各位代表！事业总是在紧跟时代中守正出新，人生总是在拥抱时代中绽放光彩。2014年春天，习近平总书记视察指导河南及开封时的殷殷嘱托，把中原及开封更加出彩与中国梦联系在一起，照亮了开封发展前行之路，点燃了550万开封人民的奋斗激情。能够赶上伟大新时代，投身开封更加出彩的宏伟事业，是我们的幸运和福分，每一个开封人都应当珍视历史际遇，切实将自己融入国家发展、民族复兴的时代潮流，对开封、对社会、对人民、对国家多一份责任和担当。

各位代表！没有脚踏实地的接续奋斗，就没有欣欣向荣的伟大事业。让我们更加紧密地团结在以习近平同志为核心的党中央周围，高举习近平新时代中国特色社会主义思想伟大旗帜，在省委、省政府和市委的坚强领导下，在市人大、市政协的监督支持下，志存高远、脚踏实地，振奋精神、锐意进取，一步一个脚印把开封的各项事业推向前进，奋力谱写新时代开封更加出彩的新篇章，以优异成绩庆祝中华人民共和国成立70周年！
